# Supplementary material for: Hot spot prediction in protein-protein interactions by an ensemble system
Source: BMC Syst Biol. 2018 Dec 31;12(Suppl 9):132. doi: 10.1186/s12918-018-0665-8 (PMC6311905; doi:10.1186/s12918-018-0665-8)
Supplement: Supplementary file 2 — All relevant MATLAB code. A simple Matlab implement of our predictor. (ZIP 19,742 kb) [file 12918_2018_665_MOESM2_ESM.zip › Additional file 2/Read ASA/ASA website/dbMPIKT/3SE8L.html]

 NetSurfP - Protein Surface Accessibility and Secondary Structure Predictions

  

|  |  |  |
| --- | --- | --- |
|  |  | NetSurfP - Protein Surface Accessibility and Secondary Structure Predictions  Technical University of Denmark |

  


---

```
# For publication of results, please cite:
# A generic method for assignment of reliability scores applied to solvent accessibility predictions.
# Bent Petersen, Thomas Nordahl Petersen, Pernille Andersen, Morten Nielsen and Claus Lundegaard
# BMC Structural Biology 2009, 9:51 doi:10.1186/1472-6807-9-51
#
# Column 1: Class assignment - B for buried or E for Exposed - Threshold: 25% exposure, but not based on RSA
# Column 2: Amino acid
# Column 3: Sequence name
# Column 4: Amino acid number
# Column 5: Relative Surface Accessibility - RSA
# Column 6: Absolute Surface Accessibility
# Column 7: Z-fit score for RSA prediction
# Column 8: Probability for Alpha-Helix
# Column 9: Probability for Beta-strand
# Column 10: Probability for Coil
E E  3SE8L                  1    0.816 142.468   0.742   0.003   0.003   0.994
B I  3SE8L                  2    0.244  45.084  -0.998   0.004   0.616   0.381
E V  3SE8L                  3    0.440  67.689   1.121   0.002   0.816   0.182
B L  3SE8L                  4    0.143  26.183   0.248   0.001   0.900   0.099
E T  3SE8L                  5    0.414  57.380   0.136   0.002   0.816   0.182
B Q  3SE8L                  6    0.153  27.237   0.569   0.004   0.514   0.481
E S  3SE8L                  7    0.354  41.501   0.028   0.004   0.138   0.858
E P  3SE8L                  8    0.477  67.729  -1.141   0.018   0.047   0.935
E G  3SE8L                  9    0.535  42.081  -1.101   0.018   0.088   0.893
E I  3SE8L                 10    0.349  64.639  -1.404   0.022   0.552   0.426
B L  3SE8L                 11    0.253  46.379  -0.147   0.018   0.846   0.136
E S  3SE8L                 12    0.483  56.643  -0.806   0.018   0.846   0.136
B L  3SE8L                 13    0.226  41.362   0.035   0.021   0.756   0.223
E S  3SE8L                 14    0.411  48.216   0.255   0.004   0.420   0.576
E P  3SE8L                 15    0.501  71.163   0.004   0.004   0.085   0.910
E G  3SE8L                 16    0.733  57.671  -0.624   0.005   0.015   0.979
E E  3SE8L                 17    0.483  84.363   0.492   0.004   0.138   0.858
E T  3SE8L                 18    0.567  78.698   1.148   0.004   0.616   0.381
B A  3SE8L                 19    0.059   6.491   0.278   0.001   0.900   0.099
E T  3SE8L                 20    0.425  58.892   1.567   0.001   0.959   0.040
B L  3SE8L                 21    0.052   9.466   0.368   0.001   0.959   0.040
E F  3SE8L                 22    0.341  68.459   0.921   0.001   0.900   0.099
B C  3SE8L                 23    0.032   4.493  -0.228   0.001   0.900   0.099
E K  3SE8L                 24    0.408  83.987   1.087   0.001   0.900   0.099
B A  3SE8L                 25    0.218  24.057  -1.360   0.002   0.816   0.182
E S  3SE8L                 26    0.326  38.231  -0.752   0.004   0.420   0.576
E Q  3SE8L                 27    0.570 101.731  -1.924   0.005   0.045   0.951
E G  3SE8L                 28    0.613  48.251  -2.283   0.005   0.015   0.979
E G  3SE8L                 29    0.494  38.901  -1.700   0.005   0.045   0.951
B N  3SE8L                 30    0.257  37.581  -0.901   0.005   0.336   0.660
B A  3SE8L                 31    0.142  15.681   0.462   0.002   0.816   0.182
B M  3SE8L                 32    0.026   5.263   0.522   0.001   0.900   0.099
B T  3SE8L                 33    0.078  10.832   0.141   0.001   0.900   0.099
B W  3SE8L                 34    0.026   6.373   0.776   0.011   0.918   0.071
B Y  3SE8L                 35    0.074  15.792   0.479   0.001   0.959   0.040
B Q  3SE8L                 36    0.128  22.897   0.535   0.001   0.900   0.099
B K  3SE8L                 37    0.138  28.428   0.356   0.003   0.718   0.279
E R  3SE8L                 38    0.411  94.188   1.751   0.004   0.085   0.910
E R  3SE8L                 39    0.557 127.622  -0.211   0.003   0.003   0.994
E G  3SE8L                 40    0.783  61.638  -2.062   0.003   0.003   0.994
E Q  3SE8L                 41    0.534  95.408   0.428   0.018   0.019   0.964
E V  3SE8L                 42    0.272  41.791  -0.754   0.018   0.047   0.935
B P  3SE8L                 43    0.141  20.050  -0.534   0.019   0.141   0.840
E R  3SE8L                 44    0.364  83.379   0.130   0.004   0.514   0.481
B L  3SE8L                 45    0.183  33.526  -0.054   0.002   0.816   0.182
B L  3SE8L                 46    0.168  30.761  -0.019   0.001   0.900   0.099
B I  3SE8L                 47    0.044   8.066  -0.985   0.001   0.959   0.040
B Y  3SE8L                 48    0.158  33.743  -0.308   0.001   0.959   0.040
B D  3SE8L                 49    0.173  24.944  -1.136   0.001   0.900   0.099
B T  3SE8L                 50    0.126  17.462  -0.942   0.021   0.451   0.528
B S  3SE8L                 51    0.293  34.293  -1.286   0.020   0.205   0.775
E R  3SE8L                 52    0.517 118.507  -1.457   0.019   0.141   0.840
E R  3SE8L                 53    0.468 107.126  -1.037   0.018   0.088   0.893
E A  3SE8L                 54    0.605  66.671  -2.562   0.018   0.047   0.935
E S  3SE8L                 55    0.671  78.606  -2.309   0.018   0.047   0.935
E G  3SE8L                 56    0.585  46.016  -2.098   0.018   0.088   0.893
B V  3SE8L                 57    0.224  34.444  -0.493   0.004   0.085   0.910
E P  3SE8L                 58    0.489  69.318  -1.028   0.004   0.085   0.910
E D  3SE8L                 59    0.554  79.788  -1.911   0.004   0.197   0.799
E R  3SE8L                 60    0.345  79.074  -0.088   0.004   0.420   0.576
B F  3SE8L                 61    0.159  31.851  -1.259   0.004   0.616   0.381
B V  3SE8L                 62    0.227  34.951  -1.789   0.004   0.616   0.381
E G  3SE8L                 63    0.339  26.687  -2.209   0.003   0.718   0.279
E S  3SE8L                 64    0.367  43.001  -1.422   0.004   0.616   0.381
E G  3SE8L                 65    0.422  33.188  -1.473   0.004   0.420   0.576
E S  3SE8L                 66    0.499  58.495  -2.050   0.005   0.262   0.733
E G  3SE8L                 67    0.568  44.678  -1.653   0.004   0.138   0.858
E T  3SE8L                 68    0.375  52.026  -0.967   0.004   0.420   0.576
E D  3SE8L                 69    0.303  43.648   0.033   0.003   0.718   0.279
B F  3SE8L                 70    0.068  13.708  -0.762   0.001   0.900   0.099
B F  3SE8L                 71    0.204  40.863   0.405   0.001   0.900   0.099
B L  3SE8L                 72    0.049   9.027  -0.004   0.001   0.900   0.099
B T  3SE8L                 73    0.229  31.804  -0.308   0.018   0.846   0.136
B I  3SE8L                 74    0.051   9.361  -0.403   0.021   0.756   0.223
E N  3SE8L                 75    0.369  54.036   0.275   0.004   0.514   0.481
E K  3SE8L                 76    0.390  80.161  -1.248   0.005   0.336   0.660
B L  3SE8L                 77    0.200  36.602  -0.242   0.019   0.141   0.840
E D  3SE8L                 78    0.442  63.663   0.590   0.018   0.088   0.893
E R  3SE8L                 79    0.445 101.974  -0.573   0.056   0.142   0.802
E E  3SE8L                 80    0.564  98.601  -2.269   0.056   0.142   0.802
E D  3SE8L                 81    0.510  73.505  -0.238   0.056   0.142   0.802
E F  3SE8L                 82    0.362  72.714  -0.875   0.021   0.279   0.699
E A  3SE8L                 83    0.379  41.755  -0.486   0.022   0.552   0.426
E V  3SE8L                 84    0.361  55.486   0.076   0.021   0.756   0.223
B Y  3SE8L                 85    0.168  35.795   1.264   0.021   0.756   0.223
B Y  3SE8L                 86    0.198  42.248   0.873   0.023   0.655   0.322
B C  3SE8L                 87    0.203  28.487  -0.717   0.021   0.451   0.528
E Q  3SE8L                 88    0.296  52.901  -0.605   0.021   0.451   0.528
B Q  3SE8L                 89    0.197  35.220  -0.567   0.021   0.451   0.528
E F  3SE8L                 90    0.362  72.673  -1.247   0.022   0.359   0.619
B E  3SE8L                 91    0.327  57.127  -1.699   0.021   0.279   0.699
E F  3SE8L                 92    0.370  74.299  -2.221   0.020   0.205   0.775
B F  3SE8L                 93    0.268  53.707  -1.601   0.018   0.088   0.893
E G  3SE8L                 94    0.349  27.498  -2.698   0.018   0.047   0.935
E L  3SE8L                 95    0.394  72.050  -1.459   0.018   0.088   0.893
E G  3SE8L                 96    0.349  27.443  -2.122   0.019   0.141   0.840
E S  3SE8L                 97    0.368  43.153  -1.476   0.020   0.205   0.775
E E  3SE8L                 98    0.343  59.940  -1.536   0.020   0.205   0.775
E L  3SE8L                 99    0.318  58.317  -1.227   0.019   0.141   0.840
E E  3SE8L                100    0.384  67.155  -1.295   0.056   0.142   0.802
E V  3SE8L                101    0.355  54.533  -1.301   0.020   0.205   0.775
E H  3SE8L                102    0.424  77.198  -1.377   0.020   0.205   0.775
E R  3SE8L                103    0.430  98.378  -1.133   0.021   0.279   0.699
E T  3SE8L                104    0.430  59.696  -0.989   0.020   0.205   0.775
E V  3SE8L                105    0.410  63.002  -1.266   0.019   0.141   0.840
E A  3SE8L                106    0.360  39.628  -0.919   0.019   0.141   0.840
E A  3SE8L                107    0.424  46.681  -1.173   0.020   0.205   0.775
E P  3SE8L                108    0.468  66.352  -1.281   0.064   0.216   0.721
E S  3SE8L                109    0.359  42.075  -0.840   0.021   0.279   0.699
B V  3SE8L                110    0.239  36.765  -1.181   0.066   0.296   0.638
E F  3SE8L                111    0.404  81.163  -0.980   0.066   0.296   0.638
E I  3SE8L                112    0.385  71.225  -1.538   0.020   0.205   0.775
E F  3SE8L                113    0.372  74.741  -1.757   0.019   0.141   0.840
E P  3SE8L                114    0.433  61.485  -1.608   0.018   0.088   0.893
E P  3SE8L                115    0.490  69.559  -1.676   0.019   0.141   0.840
E S  3SE8L                116    0.367  43.024  -1.673   0.019   0.141   0.840
E D  3SE8L                117    0.448  64.528  -1.971   0.019   0.141   0.840
E E  3SE8L                118    0.438  76.449  -2.107   0.018   0.088   0.893
E Q  3SE8L                119    0.314  56.063  -2.612   0.018   0.047   0.935
E L  3SE8L                120    0.338  61.888  -0.978   0.018   0.047   0.935
E K  3SE8L                121    0.632 129.961  -1.248   0.018   0.019   0.964
E S  3SE8L                122    0.765  89.681  -1.070   0.005   0.015   0.979
E G  3SE8L                123    0.612  48.196  -0.835   0.005   0.045   0.951
E T  3SE8L                124    0.419  58.074   0.709   0.004   0.420   0.576
B A  3SE8L                125    0.053   5.797   0.108   0.002   0.816   0.182
B S  3SE8L                126    0.189  22.127   0.810   0.001   0.900   0.099
B V  3SE8L                127    0.032   4.965   0.360   0.001   0.959   0.040
B V  3SE8L                128    0.041   6.379  -0.268   0.001   0.959   0.040
B C  3SE8L                129    0.020   2.794   0.783   0.001   0.959   0.040
B L  3SE8L                130    0.127  23.327   0.342   0.001   0.959   0.040
B L  3SE8L                131    0.026   4.852  -0.258   0.001   0.900   0.099
E N  3SE8L                132    0.270  39.528   0.299   0.002   0.816   0.182
E N  3SE8L                133    0.349  51.064  -1.006   0.004   0.420   0.576
E F  3SE8L                134    0.375  75.343  -1.416   0.005   0.262   0.733
E Y  3SE8L                135    0.344  73.598  -0.936   0.004   0.138   0.858
E P  3SE8L                136    0.447  63.458  -1.985   0.004   0.138   0.858
E R  3SE8L                137    0.584 133.828  -2.298   0.004   0.138   0.858
E E  3SE8L                138    0.390  68.063  -1.033   0.004   0.420   0.576
B A  3SE8L                139    0.095  10.480   0.118   0.002   0.816   0.182
B K  3SE8L                140    0.235  48.422   0.327   0.001   0.900   0.099
B V  3SE8L                141    0.038   5.841   0.144   0.001   0.959   0.040
B Q  3SE8L                142    0.180  32.184   0.490   0.006   0.962   0.032
B W  3SE8L                143    0.043  10.414  -0.067   0.011   0.918   0.071
E K  3SE8L                144    0.271  55.683   0.938   0.001   0.900   0.099
E V  3SE8L                145    0.380  58.467   0.558   0.003   0.718   0.279
E D  3SE8L                146    0.695 100.207   0.387   0.004   0.085   0.910
E N  3SE8L                147    0.677  99.084  -1.599   0.005   0.015   0.979
E A  3SE8L                148    0.689  75.950  -0.509   0.004   0.138   0.858
E L  3SE8L                149    0.568 104.037  -1.441   0.004   0.138   0.858
E Q  3SE8L                150    0.399  71.333  -0.411   0.004   0.138   0.858
E S  3SE8L                151    0.561  65.737  -1.269   0.004   0.197   0.799
E G  3SE8L                152    0.420  33.030  -1.780   0.005   0.262   0.733
E N  3SE8L                153    0.362  53.011  -1.800   0.004   0.420   0.576
E S  3SE8L                154    0.294  34.433  -0.996   0.003   0.718   0.279
B Q  3SE8L                155    0.277  49.401  -0.579   0.021   0.756   0.223
E E  3SE8L                156    0.510  89.097  -1.031   0.023   0.655   0.322
E S  3SE8L                157    0.309  36.215  -1.705   0.022   0.359   0.619
E V  3SE8L                158    0.360  55.301  -0.872   0.021   0.279   0.699
E T  3SE8L                159    0.418  57.990  -1.034   0.020   0.205   0.775
E E  3SE8L                160    0.386  67.399  -0.670   0.019   0.141   0.840
E Q  3SE8L                161    0.526  93.997  -1.277   0.020   0.205   0.775
E D  3SE8L                162    0.598  86.201  -2.499   0.019   0.141   0.840
E S  3SE8L                163    0.603  70.718  -1.546   0.018   0.047   0.935
E K  3SE8L                164    0.513 105.504  -1.021   0.018   0.019   0.964
E D  3SE8L                165    0.673  97.023  -1.770   0.005   0.015   0.979
E S  3SE8L                166    0.434  50.818  -1.324   0.005   0.045   0.951
E T  3SE8L                167    0.305  42.331   0.377   0.004   0.420   0.576
B Y  3SE8L                168    0.065  13.826  -0.554   0.003   0.718   0.279
B S  3SE8L                169    0.084   9.892  -1.315   0.002   0.816   0.182
B L  3SE8L                170    0.057  10.437  -1.023   0.001   0.900   0.099
B S  3SE8L                171    0.121  14.228  -0.500   0.001   0.959   0.040
B S  3SE8L                172    0.038   4.454  -1.636   0.001   0.959   0.040
B T  3SE8L                173    0.186  25.770   0.127   0.001   0.959   0.040
B L  3SE8L                174    0.040   7.251  -0.118   0.001   0.900   0.099
E T  3SE8L                175    0.305  42.290   0.774   0.001   0.900   0.099
B L  3SE8L                176    0.054   9.887   0.663   0.003   0.718   0.279
E S  3SE8L                177    0.338  39.637   1.453   0.005   0.336   0.660
E K  3SE8L                178    0.477  98.222  -0.495   0.019   0.141   0.840
E A  3SE8L                179    0.611  67.332  -0.904   0.019   0.141   0.840
E D  3SE8L                180    0.537  77.411   0.889   0.020   0.205   0.775
B Y  3SE8L                181    0.186  39.748  -0.712   0.018   0.088   0.893
E E  3SE8L                182    0.682 119.215   0.316   0.005   0.045   0.951
E K  3SE8L                183    0.612 125.888   0.537   0.005   0.045   0.951
E H  3SE8L                184    0.622 113.105  -0.753   0.005   0.045   0.951
E K  3SE8L                185    0.550 113.032   0.236   0.005   0.262   0.733
E V  3SE8L                186    0.455  69.980   1.740   0.002   0.816   0.182
B Y  3SE8L                187    0.062  13.164   0.967   0.001   0.959   0.040
E A  3SE8L                188    0.314  34.636   1.684   0.001   0.959   0.040
B C  3SE8L                189    0.023   3.173   0.822   0.001   0.959   0.040
B E  3SE8L                190    0.267  46.715   1.331   0.001   0.959   0.040
B V  3SE8L                191    0.067  10.313  -0.021   0.001   0.959   0.040
E T  3SE8L                192    0.424  58.809   0.675   0.001   0.959   0.040
B H  3SE8L                193    0.139  25.357   0.480   0.002   0.816   0.182
E Q  3SE8L                194    0.629 112.304   0.016   0.005   0.336   0.660
E G  3SE8L                195    0.611  48.117  -1.592   0.004   0.085   0.910
E L  3SE8L                196    0.421  77.030  -0.550   0.005   0.045   0.951
E S  3SE8L                197    0.658  77.106  -0.203   0.005   0.045   0.951
E S  3SE8L                198    0.530  62.104   0.086   0.019   0.141   0.840
E P  3SE8L                199    0.464  65.898   0.201   0.005   0.336   0.660
B V  3SE8L                200    0.178  27.374  -0.180   0.003   0.718   0.279
E T  3SE8L                201    0.545  75.591   0.370   0.001   0.900   0.099
E K  3SE8L                202    0.274  56.465   1.266   0.001   0.900   0.099
E S  3SE8L                203    0.458  53.701   0.230   0.001   0.900   0.099
B F  3SE8L                204    0.101  20.351   0.862   0.001   0.900   0.099
E N  3SE8L                205    0.512  74.986   1.668   0.003   0.718   0.279
E R  3SE8L                206    0.432  98.905   0.567   0.020   0.205   0.775
E G  3SE8L                207    0.639  50.258  -1.248   0.019   0.141   0.840
E E  3SE8L                208    0.677 118.254   1.633   0.018   0.088   0.893
E C  3SE8L                209    0.513  72.081  -1.266   0.003   0.003   0.994

Explain the output.  Go back.
```

---
